# HoLEP versus ThuFLEP: perioperative and functional outcomes in prostate enucleation

**DOI:** 10.1007/s10103-026-04857-w

**Published:** 2026-04-24

**Authors:** Ersin Gokmen, Turker Altuntas, Mohammad Yasir Sahak, Hamza Tunahan Midilli, Gunal Ozgur, Kamil Cam, Tufan Tarcan, Tarik Emre Sener

**Affiliations:** 1 Deparment of Urology, Marmara Üniversitesi Eğitim ve Araştırma Hastanesi, Istanbul, Turkey; 2 Department of Urology, School of Medicine, Marmara University, Istanbul, Turkey; 3 Department of Urology, School of Medicine, Koç University, Istanbul, Turkey

**Keywords:** Prostate, Laser, Resection

## Abstract

**Objective:**

To compare the outcomes of laser enucleation techniques by evaluating the postoperative data of patients with benign prostate obstruction (BPO) undergoing ThuFLEP and HoLEP.

**Methods:**

This retrospective study included men > 50 years with LUTS due to BPO who underwent HoLEP or ThuFLEP by a single experienced surgeon between January 2023 and January 2024. Patients were also stratified into subgroups according to prostate size (< 80 g vs. ≥ 80 g). The primary outcome was to compare success rate, operative duration, and efficiency of HoLEP and ThuFLEP; secondary outcomes included safety profile, catheter removal time, and long-term complications (stress urinary incontinence and stricture).

**Results:**

A total of 163 patients (HoLEP: *n* = 94, ThuFLEP: *n* = 69) were included. Median operation time was longer in ThuFLEP (100 vs. 90 min, *p* = 0.001). Enucleation ratio, surgical efficiency, haemoglobin drop, ∆PVR, ∆Qmax, and ∆QoL were similar. IPSS improvement was greater in ThuFLEP (15 vs. 10 points, *p* = 0.023). In prostates > 80 g, ThuFLEP had longer operation and hospitalization times, but other perioperative and functional outcomes were comparable. Complication rates, catheterization time, and discharge duration were similar in all groups.

**Conclusions:**

Both HoLEP and ThuFLEP are safe and effective for BPH treatment. ThuFLEP shows slightly longer operative time, especially in larger prostates, while functional improvements and complication rates are comparable.

## Introduction

Benign prostatic hyperplasia (BPH) is a common cause of lower urinary tract symptoms (LUTS) in aging men, with a major impact on quality of life (QoL) [1, 2]. Approximately 50% of men during the 7th decade and 80% during 9th decade present LUTS. This patient group constitutes a significant proportion of urology outpatient clinic admissions.

Conservative, medical, and surgical approaches are at the forefront in the treatment of patients with BPH. Accepted medical treatments for moderate to severe LUTS are alpha-blockers and 5-alpha-reductase inhibitors, in single, or combination therapy [3].

As surgical approaches, resection, open or laser enucleation, vaporization, and minimally invasive techniques are recommended in current guidelines [4]. Transurethral resection of prostate (TURP) is recommended for moderately enlarged prostates of up to 80 cm3, while larger prostates have traditionally been treated with open simple prostatectomy (OP) [4] which is effective but carries a risk of considerable perioperative morbidity [5]. Among these surgical options, laser enucleation techniques have recently come to the forefront compared to other methods due to their advantages.

Since the introduction of the anatomical endoscopic enucleation of the prostate (AEEP) into the armamentarium of benign prostatic obstruction (BPO) treatment has been proven to be a minimally invasive, size-independent method in numerous randomized controlled trials (RCTs) with excellent long-term results [6, 7]. When surgical options within the AEEP framework are reviewed, it is evident from the literature that techniques using holmium: YAG (Ho: YAG) and thulium laser systems have been well established and widely used over many years [8]. HoLEP has been recommended by the current guidelines of the European Association of Urology (EAU) in men with substantially enlarged prostates (e.g. > 80 cc) [4]. Another type of laser enucleation, ThuFLEP, also constitutes an alternative to HoLEP in terms of complication rates and feasibility [9]. Like HoLEP, it has shown excellent efficacy in improving patients’ micturition and QoL, with the same advantages over TURP and OP [6, 9].

Comparative data between HoLEP and ThuFLEP regarding perioperative outcomes and postoperative functional results are limited, and direct comparative studies between these two modalities are relatively scarce. However, differences exist in the laser effects on the tissue which may be reflected into clinical practice with differences in various outcomes. Existing studies often include heterogeneous patient populations and frequently lack size-stratified subgroup analyses. In this study, we aimed to present our own data on the perioperative and functional outcomes of HoLEP and ThuFLEP in patients with BPH. We also sought to investigate the potential clinical impact of laser-specific characteristics on surgical performance.

## Methods

This study was conducted at our tertiary university hospital. Data from patients who underwent HoLEP and ThuFLEP surgeries performed by a single experienced surgeon between January 2023 and January 2024 were retrospectively reviewed. The study was carried out in compliance with the protocol and Good Clinical Practice, as described in: ICH Harmonized Tripartite Guidelines for Good Clinical Practice 1996; the Declaration of Helsinki, concerning medical research in humans (Recommendations Guiding Physicians in Biomedical Research Involving Human Subjects, Helsinki 1964, amended Tokyo 1975, Venice 1983, Hong Kong 1989, Somerset West 1996). The study was approved by the Institutional Review Board (IRB) where the study took place with the protocol number of Marmara University Local Ethics Committee 09.2025.714.

Eligible patients were men > 50 years of age presenting to our outpatient clinic with LUTS attributed to BPO with an indication for surgical treatment according to EAU Guidelines on Non-Neurogenic Male LUTS [4]. Patients with a prior history of BPE surgery, under anticoagulant therapy, history of prostate cancer diagnosis were excluded. All surgical procedures were performed by an experienced surgeon who had performed at least 100 HoLEP and ThuFLEP procedures. All the patients received prophylactic antibiotics according to the preoperative urine culture results. All the urinary tract infections were treated prior to surgery. Patients with stable vital signs, no significant haematuria, safe catheter removal, and spontaneous micturition were discharged. Patients with a minimum of 12 months of postoperative follow-up were included in the study.

All procedures were carried out under general or spinal anaesthesia. A 26 F continuous-flow laser resectoscope (R. Wolf) was used for both procedures. Enucleation was performed using Quanta System laser platforms: a 60-W Ho: YAG laser (Cyber Ho 60) for HoLEP and a 60-W thulium fiber laser (Fiber Dust 60 W) for ThuFLEP, with all procedures carried out using a 550-µm laser fiber. The choice of laser system to be used in each patient depended on the availability of the device in the operating room on the day of operation. To provide procedural consistency, laser settings were standardized across all patients. For both laser systems, the energy parameters were set at 2 Joules and 30 Hz, resulting in a total power output of 60 W, and the Virtual Basket pulse modulation mode was used throughout the procedures. All enucleations were performed using the en bloc technique. Following enucleation, the prostate tissue was removed using a Hawk morcellator. Normal saline solution was used for irrigation during the procedures. At the end of the operation, a 22-Fr three-way Foley catheter was inserted, and continuous bladder irrigation was started before transferring the patients to the ward.

All assessments were carried out using a self-calibrating automated uroflowmetry system (Aymed^®^ Uroscan Plus) and a pelvic ultrasound device equipped for PVR measurement (Siemens^®^ Acuson X300). Uroflowmetry tests were conducted in accordance with the International Continence Society (ICS) recommendations [10]. The maximum flow rate (Qmax) values of all patients were recorded. PVR urine volume was determined immediately after voiding via pelvic ultrasonography, using the formula: “anteroposterior diameter × oblique diameter × transverse diameter × 0.52” [11]. All patients were evaluated using the validated International Prostate Symptom Score (IPSS), and the corresponding Quality of Life (QoL) score was recorded.

Patients who underwent HoLEP were classified as Group 1, and those who underwent ThuFLEP were classified as Group 2. Initially, the two groups were compared in terms of demographic characteristics, perioperative data, changes in preoperative and postoperative lower urinary tract parameters, enucleated prostate volumes, hospitalization and catheter removal times, pathological findings, and complications. Subsequently, these comparisons were repeated by separately analysing patients with prostate volumes below and above 80 g.

Postoperative functional outcomes, including uroflowmetry parameters, PVR, and symptom scores were assessed at the 1-month postoperative follow-up and compared with preoperative values. Postoperative complications were evaluated during follow-up visits at 1, 6, and 12 months. During postoperative follow-up, while evaluating postoperative complications, patients presenting with obstructive voiding symptoms and demonstrating low Qmax and an obstructive pattern on uroflowmetry were diagnosed with stricture based on cystourethroscopic findings. Postoperative stress urinary incontinence was defined as stress-type urine leakage persisting for at least 6 months after surgery, confirmed either by clinical assessment or standardized pad test [12].

The primary objective was to compare the success rate, operative duration, and efficiency of HoLEP and ThuFLEP for BPO. The secondary objective was to compare the safety profile, catheter removal time and the long-term outcomes such as incontinence rates and stricture rates of the same subgroups of patients in the same cohort.

### Statistical analysis

Statistical analyses were conducted using IBM SPSS 25.0. Due to non-normal data distribution (Kolmogorov-Smirnov test), non-parametric tests were applied. Numerical data were presented as median interquartile range (IQR), categorical data as counts/percentages. The Chi-square test analysed categorical data, while the Mann-Whitney tests compared groups. A p-value < 0.05 was considered significant.

## Results

A total of 163 patients were included in the study. The mean age of the patients was 67.12 ± 7.02 years. The median prostate volume was 85 (60–115) g. Of the 163 patients, 94 (57.7%) underwent HoLEP (Group 1) and 69 (42.3%) underwent ThuFLEP (Group 2).

Initially, demographic data were compared between groups. The mean age was similar. The median prostate volume did not differ significantly between the groups. The median operation time was significantly longer in Group 2 compared to Group 1 (100 vs. 90 min, *p* = 0.001). Although the enucleated prostate volume was lower in Group 2, the difference was not statistically significant (*p* = 0.164). Enucleation ratio, surgical efficiency, haemoglobin drop, ∆PVR, ∆Qmax, and ∆QoL were comparable between groups (all *p* > 0.05). However, the improvement in IPSS score was significantly greater in Group 2 (15 vs. 10 points, *p* = 0.023) (Table 1).

**Table 1:** Comparison of Demographic Data and Outcomes

	Group 1 (*n*:94) HoLEP	Group 2 (*n*:69) ThuFLEP	*P* value
Age (year) mean (std)	67.05 +/- 6.44	66.74 +/- 8.21	0.86
Prostate Volume (gr) median (IQR)	84.5 (60-114.25)	90 (60–122)	0.647
PSA (ng/dl) median (IQR)	4.1 (2.34–6.51)	3.2 (1.74–5.11)	0.078
Operation Time (minute) median (IQR)	90 (70–110)	100 (90–141)	**0.001**
Enucleated Prostate Volume (gr) median (IQR)	62.5 (40-81.25)	50 (34.25–73.75)	0.164
Enucleation Ratio (Enucleated volume / prostate volume) (gr/gr) median (IQR)	0.71 (0.59–0.83)	0.63 (0.46–0.75)	0.112
Surgical Efficiency (Enucleated volume / operation duration) (gr/min) median (IQR)	0.54 (0.33–0.66)	0.41 (0.29–0.53)	0.077
∆Hgb (g/dL) median (IQR)	0.9 (0.3–1.4)	0.9 (0.3–1.4)	0.982
∆PVR (mL/s) median (IQR)	108.5 (40–231)	99.5 (51.25–204)	0.635
∆Qmax (mL/s) median (IQR)	10 (6-15.5)	9 (3.5–15)	0.397
∆IPSS median (IQR)	10 (3.5–15.5)	15 (8.75-18)	**0.023**
∆QoL median (IQR)	2 (1.25-3)	3 (1.5-4)	0.222

*Hgb* Haemoglobin, *IPSS* International Prostate Symptom Score, *IQR* Interquartile Range, *PVR* Postvoid Residual, *PSA* Prostate-Specific Antigen, *Qmax* Maximum Flow Rate, *QoL* Quality of Life

Regarding intraoperative complications, no significant differences were observed between the groups in terms of capsular perforation, sub trigonal undermining, bladder mucosal injury, or bladder perforation. Catheterization time and hospitalization duration did not differ significantly. At the 1st, 6th, and 12th postoperative months, the rates of delayed bleeding, stress urinary incontinence, and urethral stricture were comparable between groups. There was no significant difference between the preoperative and postoperative total PSA values in patients who underwent enucleation surgery with either surgical technique (Table 2).

**Table 2:** Comparison of Complications

		Group 1 (*n*:94) HoLEP	Group 2 (*n*:69) ThuFLEP	*P* value
Intraoperative Complications n (%)	Capsular Perforation	6 (6.4)	4 (5.8)	0.836
	Sub trigonal Undermining	6 (6.4)	5 (7.2)	
	Bladder Mucosal Injury	2 (2.1)	3 (4.3)	
	Bladder Perforation Requiring Open Repair	1 (1.1)	0	
Clavien-Dindo Complications n (%)	CD 1	6 (6.4)	10 (14.5)	0.418
	CD 2	7 (7.4)	5 (7.2)	
	CD 3	1 (1.1)	0	
	CD 4	1 (1.1)	0	
Postoperative Prolonged Bleeding Before Catheter Removal n (%)		2 (2.2)	1 (1.4)	0.743
Postoperative Catheter Retrieval (day) median (IQR)		2 (2–3)	2 (2–3)	0.887
Discharge (day) median (IQR)		3 (2–3)	3 (2–3)	0.493
Postoperative PSA (ng/dl) median (IQR)		0.66 (0.2–1.13)	0.5 (0.22–1.11)	0.76
Postoperative Complications n (%)				
1st month	SUI	18 (19.1)	15 (21.7)	0.455
	Urethral stricture	0	1 (1.4)	
6th month	SUI	6 (6.4)	6 (8.7)	0.102
	Urethral stricture	0	3 (4.3)	
12th month	SUI	4 (4.3)	4 (5.8)	0.109
	Urethral stricture	0	3 (4.3)	

*IQR *Interquartile Range, *PSA* Prostate-Specific Antigen, *Qmax* Maximum Flow Rate, *SUI* Stress Urinary Incontinence

When subgroup analyses were performed according to prostate size, in prostates > 80 g (*n* = 92), Group 2 had a significantly longer median operation time (120 vs. 95 min, *p* = 0.001). Discharge time was also significantly longer in Group 1 (median 3 vs. 2 days, *p* = 0.036). Other perioperative parameters, complications, and functional outcomes were not significantly different (Table 3).

**Table 3:** Comparison of Surgeries for > 80 gr Prostates

	Group 1 (*n*:54) HoLEP	Group 2 (*n*:38) ThuFLEP	*p* value
Age (years) mean (std)	67.33 +/- 7.21	67.21 +/- 6.55	0.486
Prostate Volume (gr) median (IQR)	108 (90-132.5)	110 (99.5–150)	0.17
PSA (ng/dl) median (IQR)	5.07 (3.14–6.88)	4.12 (2.33–6.73)	0.307
Operation Time (minute) median (IQR)	95 (81.25–120)	120 (95–150)	**0.001**
Enucleated Prostate Volume (gr) median (IQR)	80 (60–90)	70 (60–80)	0.318
Enucleation Ratio (Enucleated volume / Prostate Volume) (gr/gr) median (IQR)	0.64 (0.58–0.82)	0.64 (0.45–0.76)	0.667
Surgical Efficiency (Enucleated Volume / Operation Duration) (gr/minute) median (IQR)	0.6 (0.43–0.85)	0.46 (0.41–0.59)	0.164
∆Hgb median (IQR)	1.1 (0.55–1.7)	1 (0.37–1.92)	0.664
∆PVR median (IQR)	130 (60–260)	90 (49-126.25)	0.104
∆Qmax median (IQR)	10.5 (6-17.25)	9.5 (4–15)	0.292
∆IPSS median (IQR)	12 (8–16)	16 (10–18)	0.124
∆QoL median (IQR)	3 (2–3)	3 (2–4)	0.15
Postoperative Catheter Retrieval (day) median (IQR)	3 (2–3)	2 (2–3)	0.407
Discharge (day) median (IQR)	3 (2–3)	2 (2–3)	**0.036**

*Hgb* Haemoglobin, *IPSS* International Prostate Symptom Score, *IQR* Interquartile Range, *PVR* Postvoid Residual, *PSA* Prostate-Specific Antigen, *Qmax* Maximum Flow Rate, *QoL* Quality of Life

In patients with prostates < 80 g (*n* = 71), demographic characteristics and perioperative outcomes were similar between groups, except for the enucleation ratio, which was significantly higher in Group 1 (0.73 vs. 0.53, *p* = 0.045). No significant differences were found regarding intraoperative or postoperative complications during follow-up for smaller prostate group (Table 4).

**Table 4:** Comparison of Surgeries for < 80 gr Prostates

	Group 1 (*n*:40) HoLEP	Group 2 (*n*:31) ThuFLEP	*p* value
Age (years) mean (std)	67.05 +/- 6.44	66.74 +/- 8.21	0.86
Prostate Volume (gr) median (IQR)	58 (46–68)	56 (50–70)	0.602
PSA (ng/dl) median (IQR)	3.04 (1.61–4.47)	1.9 (0.89–3.69)	0.116
Operation Time (minute) median (IQR)	75 (60–90)	90 (55–120)	0.116
Enucleated Prostate Volume (gr) median (IQR)	41 (30–52)	34 (20-42.5)	0.193
Enucleation Ratio (Enucleated volume / Prostate Volume) (gr/gr) median (IQR)	0.73 (0.66–0.94)	0.53 (0.47–0.79)	**0.045**
Surgical Efficiency (Enucleated Volume / Operation Duration) (gr/minute) median (IQR)	0.34 (0.31–0.55)	0.28 (0.22–0.36)	0.163
∆Hgb median (IQR)	0.8 (0.2–1.3)	0.6 (0.2–1.2)	0.807
∆PVR median (IQR)	100 (36–210)	124.5 (51.25–204)	0.286
∆Qmax median (IQR)	10 (6–14)	8 (3–16)	0.802
∆IPSS median (IQR)	6.5 (1–12)	10.5 (3.75–18.25)	0.097
∆QoL median (IQR)	2 (1–3)	2 (1–3)	0.936
Postoperative Catheter Retrieval (day) median (IQR)	2 (2–3)	3 (2–3)	0.508
Discharge (day) median (IQR)	2.5(2–3)	3 (2–4)	0.274

*Hgb* Haemoglobin, *IPSS* International Prostate Symptom Score, *IQR* Interquartile Range, *PVR* Postvoid Residual, *PSA* Prostate-Specific Antigen, *Qmax* Maximum Flow Rate, *QoL* Quality of Life

Incidental prostate cancer was detected in 6 patients who underwent enucleation surgery. Among these, 5 patients (83.3%) underwent HoLEP, and 1 patient (16.7%) underwent ThuFLEP. The pathology results revealed Gleason 3 + 3 in 5 patients and Gleason 3 + 4 in 1 patient. All patients diagnosed with incidental prostate cancer received a combination of radiotherapy and hormone therapy as treatment. The pathology results of all other patients were benign.

## Discussion

In daily clinical practice, surgical approaches are among the treatment options we resort to in patients with BPH, a patient population we frequently encounter, when appropriate indications are present. With the advancement of laser technology, enucleation surgeries, particularly HoLEP and ThuFLEP, have gained popularity. In this study, we aimed to compare the outcomes of these two techniques by evaluating the data of patients who underwent these surgeries with a minimum postoperative follow-up of one year. A key feature of this study is the comparison of low-power HoLEP and low-power thulium fiber laser techniques. The use of low-power lasers may offer potential advantages such as more controlled energy delivery, limited thermal damage, and better hemostasis [13, 14]. Therefore, low-power enucleation techniques are gaining increasing attention in clinical practice.

In this study, we compared perioperative and functional outcomes of HoLEP and ThuFLEP in 163 patients. Overall, both techniques demonstrated comparable demographic characteristics, complication rates, and functional outcomes. ThuFLEP was associated with a significantly longer operative time, and although the enucleated prostate volume was lower, this did not reach statistical significance. Notably, the improvement in IPSS was greater in the ThuFLEP group. Although the underlying mechanism of this finding cannot be definitively established, we believe that certain technical and laser-related factors may have contributed to this outcome. thulium fiber laser systems operate at a wavelength that is highly absorbed by water, providing a more continuous and precise cutting profile along with effective coagulation. The longer operating times and lower enucleation volumes observed in the ThuFLEP group may be related to the laser’s more superficial effect profile and more controlled cutting feature. Additionally, the more precise dissection achieved with thulium fiber laser may have led to more conservative tissue removal. These characteristics may facilitate smoother dissection planes and, consequently, contribute to a more pronounced early improvement in symptom scores. Despite a lower enucleation volume in the ThuFLEP group, a greater improvement in IPSS was observed. This finding may appear contradictory, as symptom relief is generally expected to correlate with the amount of tissue removed. However, functional outcomes are not solely dependent on enucleation volume. The more precise and uniform dissection planes achieved with thulium fiber laser may allow for better preservation of the external sphincter and surrounding structures, potentially reducing postoperative edema and irritation. In addition, the lower depth of penetration and reduced thermal damage associated with thulium fiber laser may contribute to decreased irritative symptoms, thereby improving early postoperative functional outcomes. Nevertheless, these findings should be interpreted with caution, as early functional improvement may not directly reflect long-term outcomes. Further prospective studies with longer follow-up are required to clarify this relationship.

Subgroup analyses revealed that for prostates > 80 g, ThuFLEP was associated with longer operative and hospitalization times, while in prostates < 80 g, HoLEP achieved a higher enucleation ratio. When considering factors that may have influenced this result, we believe that in larger prostates, the thulium fiber laser may lead to a slower enucleation pace compared with the pulsed energy delivery of the Ho: YAG laser. From a technical perspective, Ho: YAG lasers provide effective mechanical tissue separation and reliable hemostasis through pulsed energy delivery with shallow tissue penetration, whereas thulium fiber lasers offer more homogeneous energy distribution, higher cutting precision, and enhanced coagulative properties [13]. These laser-specific characteristics may contribute to clinically meaningful differences in surgical workflow, enucleation speed, and early postoperative functional recovery. Given the limited availability of high-quality literature focusing on laser physics and tissue interaction within the context of AEEP, our study provides additional insight into how laser-related properties may translate into clinical outcomes. Although both HoLEP and ThuFLEP remain effective and safe enucleation techniques, a better understanding of these technical nuances may facilitate the individualization of surgical approach based on patient characteristics and targeted perioperative outcomes.

When the publications on the literature are considered, there are studies comparing thulium and holmium laser technologies in BPH surgery, similar to our work. In the prospective study designed by Kosiba et al., 150 patients with BPO were randomly divided into two groups, with the first group undergoing ThuFLEP and the second group HoLEP. At the 3-month follow-up, no differences were observed in terms of IPSS and QoL. Residual urine, Qmax, complication rates, and operative parameters were also found to be similar between the groups. This study, similar to ours, demonstrates that both surgical techniques offer comparable functional outcomes [15].

In the multicentre study by Gauhar et al. including 4,216 BPH patients, the surgical and functional enucleation outcomes of HoLEP and ThuFLEP were compared across different prostate sizes. Although total operative time was similar, enucleation and morcellation times were longer in the ThuFLEP group. Similar to our study, postoperative incontinence rates were comparable between the groups. Postoperative acute urinary retention was higher in the ThuFLEP group. Regarding functional outcomes, Qmax was higher and PVR was lower in the ThuFLEP group. Improvements in IPSS and quality of life were similar in both groups [16].

In the study designed by Enikeev et al. with 163 patients (77 HoLEP, 86 ThuFLEP), complication rates, catheterization time, and length of hospital stay were found to be similar between the groups. No differences were observed in functional outcomes up to 6 months. No differences were noted regarding complications either. Although this randomized prospective study had a relatively short follow-up period of 6 months, it enriched the evaluation by including self-administered questionnaires such as the IPSS, the International Index of Erectile Function-5 (IIEF-5), the Questionnaire for Urinary Incontinence Diagnosis (QUID), and the Incontinence Questionnaire Male Lower Urinary Tract Symptoms Module (ICIQ-MLUTS). The study demonstrated that there was no significant difference between the two surgical techniques in terms of postoperative irritative symptoms [17].

A review of the literature shows that there are publications comparing holmium laser enucleation with thulium: YAG laser enucleation. In the study by Zhang et al., 133 patients were included, with 71 patients undergoing ThuLEP and 62 undergoing HoLEP. The operation time was found to be longer in patients who underwent ThuLEP. In this study, which also evaluated blood loss, ThuLEP was found to cause less bleeding. In both methods, catheterization time, symptom scores, and uroflow parameters improved similarly [18].

In the study by Pirola et al., which retrospectively evaluated 117 patients undergoing HoLEP or ThuFLEP, enucleation time was found to be longer in the HoLEP group. Catheterization duration was similar between the two groups. In both methods, IPSS and QoL scores significantly decreased, and maximum urinary flow rate increased. In this study, HoLEP was superior to ThuFLEP in terms of 1-year PSA reduction. In our study, no difference was observed between the two techniques in terms of PSA reduction [19].

In the study published by Zhang et al. in 2020, the perioperative and functional outcomes of HoLEP and ThuFLEP for the treatment of large-volume benign prostatic hyperplasia were compared. In both methods, morcellation time, resected tissue weight, haemoglobin loss, catheterization time, and hospital stay were similar. Changes in IPSS, QoL, Qmax, PVR, and PSA at the 18-month follow-up were also similar. Although not statistically significant, the operation and enucleation times were shorter in the ThuFLEP group. This discrepancy may be attributed to differences in surgeon experience, patient selection, and study design. Both studies, however, consistently demonstrate that HoLEP and ThuFLEP are highly effective and safe techniques for relieving lower urinary tract symptoms, and the differences between the two methods are clinically negligible [20].

This study has several limitations. First, its retrospective and single-centre design may limit the generalizability of the findings. Second, the follow-up period of 12 months is relatively short to evaluate long-term functional outcomes and complications such as urethral stricture. Third, absence of randomization in the choice of laser modality. HoLEP and ThuFLEP were performed according to surgeon experience and the availability of the devices in the operating room rather than predefined patient-specific criteria, which may have introduced a degree of selection bias. In addition, we consider the lack of evaluation and comparison of erectile function between these two types of enucleation surgeries to be a limitation. In this study, energy levels were standardized by using similar power settings in the HoLEP and ThuFLEP groups. However, although energy standardization was attempted, the mechanisms of interaction with tissue differ between these two laser technologies, and the optimal operating parameters may vary for each system. We would like to point out that this situation is a limitation of the study and should be taken into account when interpreting the results. Despite these limitations, the study provides valuable insight into the comparative safety and efficacy of HoLEP and ThuFLEP in clinical practice.

## Conclusion

Both HoLEP and ThuFLEP procedures provided safe and effective results, with comparable intraoperative parameters, complications, and functional outcomes. ThuFLEP was associated with a longer operative time, particularly in prostates > 80 g, and slightly longer hospitalization. Improvements in IPSS, QoL, Qmax, and PVR were similar between groups, although IPSS improvement was greater in ThuFLEP overall. Subgroup analysis indicated that prostate size may influence operative time and enucleation ratio but did not significantly affect complication rates or functional outcomes.

## Data Availability

Data available on request due to privacy/ethical restrictions.
